# A Risk Score to Diagnose Cardiac Involvement and Provide Prognosis Information in Patients at Risk of Cardiac Light-Chain Amyloidosis

**DOI:** 10.3389/fcvm.2022.817456

**Published:** 2022-03-09

**Authors:** Yan Wu, Cailing Pu, Wenchao Zhu, Chengbin He, Jingle Fei, Hongjie Hu

**Affiliations:** ^1^Department of Radiology, Sir Run Run Shaw Hospital, Zhejiang University School of Medicine, Hangzhou, China; ^2^Department of Radiology, Lishui Central Hospital, Lishui, China

**Keywords:** low QRS voltage, pericardial effusion, strain, prognosis, diagnosis, cardiac amyloidosis -AL type, late gadolinium enhanced imaging

## Abstract

**Background:**

Cardiac light-chain amyloidosis (AL CA) portends poor prognosis. Contrast cardiac magnetic resonance (CMR) with late gadolinium enhancement (LGE) imaging is an important tool in recognizing AL CA. But contraindications to contrast CMR would significantly restrict its clinical application value. Our study aims to construct a convenient risk score to help identify cardiac involvement in patients at risk of AL CA. Moreover, we also investigate whether this risk score could provide prognosis information.

**Materials and Methods:**

Sixty-three patients at risk of AL CA were retrospectively included in our study. Basic clinical characters, lab results, 12-lead electrocardiogram data, and cardiac magnetic resonance image data were collected. AL CA was diagnosed according to typical CA LGE pattern. Logistic analysis was used to figure out predictive parameters of AL CA and their β coefficients, further constructing the risk score. Receiver operating characteristics (ROC) curve was used to find the cut-off point best distinguishing AL CA+ from AL CA–patients. Bootstrapping was used for internal validation. All patients were divided into high-risk and low-risk group according to the diagnostic cut-off point, and followed up for survival information. Kaplan-Meier plots and log-rank test were performed to analyze if this score had prognostic value.

**Results:**

The risk score finally consisted of 4 parameters: pericardial effusion (PE) (1 point), low electrocardiographic QRS voltages (LQRSV) (1 point), CMR-derived impaired global radial strain (GRS) (<15.14%) (1 point) and increased left ventricular maximum wall thickness (LVMWT) (>13 mm) (2 points). Total score ranged from 0 to 5 points. A cut-off point of 1.5 showed highest accuracy in diagnosing AL CA with an AUC of 0.961 (95% CI: 0.924–0.997, sensitivity: 90.6%, specificity: 83.9%). Kaplan-Meier plots and log-rank test showed that the high-risk group had significantly poor overall survival rates.

**Conclusion:**

In patients at risk of AL CA, a risk score incorporating the presence of PE, LQRSV, and CMR-derived impaired GRS and increased LVMWT is predictive of a diagnosis of AL CA by LGE criteria. This risk score may be helpful especially when contrast CMR is not available or contraindicated, and further studies should be considered to validate this score.

## Introduction

Light-chain amyloidosis (AL) is the most common type of systemic amyloidosis (1). Abnormal monoclonal plasma cell proliferation is the major cause of AL amyloidosis and on rare occasion, malignant lymphoproliferations would also lead to this disease (2, 3). Multi-organ involvement is one of the characteristics of AL amyloidosis, which makes the therapy complicated.

AL CA was proved to affect prognosis most, constituting the major cause of death (4). It is reported that, without treatment, the median survival time of AL CA patients was 6 months since the presentation of symptoms (5). However, atypical symptoms in the early stage and deficient recognition of cardiac involvement make AL CA an underdiagnosed disease (6). Therefore, the majority of AL CA patients receive their initial specific treatment at the late stage, which has limited contribution to sound prognosis. Early detection of AL CA may alter the therapy protocol, potentially altering the prognosis (7).

Endomyocardial biopsy (EMB) is the reference standard to diagnose AL CA. It could provide an access to pathology result. Despite the edge of EMB, the clinical application of EMB is greatly restricted by its invasive nature, technical difficulties, and potential sample error (8). Actually, imaging methods are more widely applicated than EMB, for they allow for noninvasive and repeatable global evaluation of cardiac amyloidosis burden. Contrast cardiac magnetic resonance (CMR) with Late-gadolinium-enhancement (LGE) imaging is a commonly used noninvasive imaging method to diagnose and evaluate cardiac amyloidosis (CA). For AL CA patients, amyloidosis deposition increases extracellular volume (ECV) of myocardium, which leads to the wash-out delay of contrast agent in the lesion area compared to normal myocardial tissue (9), resulting in increased signal intensity on T1-weighted imaging. While the characteristic LGE distribution shows high accuracy in diagnosing cardiac involvement (10), the high occurrence rate of renal dysfunction among patients at risk of AL CA may render these patients unsuitable candidates for LGE-imaging (11). In addition, the side effects of gadolinium injection have been discussed before (12, 13). Hence, effective and widely applicable method is still urgently desired to facilitate the diagnosis of AL CA.

In our study, we aimed to construct a risk score helping discover cardiac involvement in patients with risk of AL CA, especially in those with contraindications to contrast CMR scan. Furthermore, we also tested the ability of this risk score in prognosticating outcomes of these patients.

## Materials and Methods

### Patients

**Figure 1:**
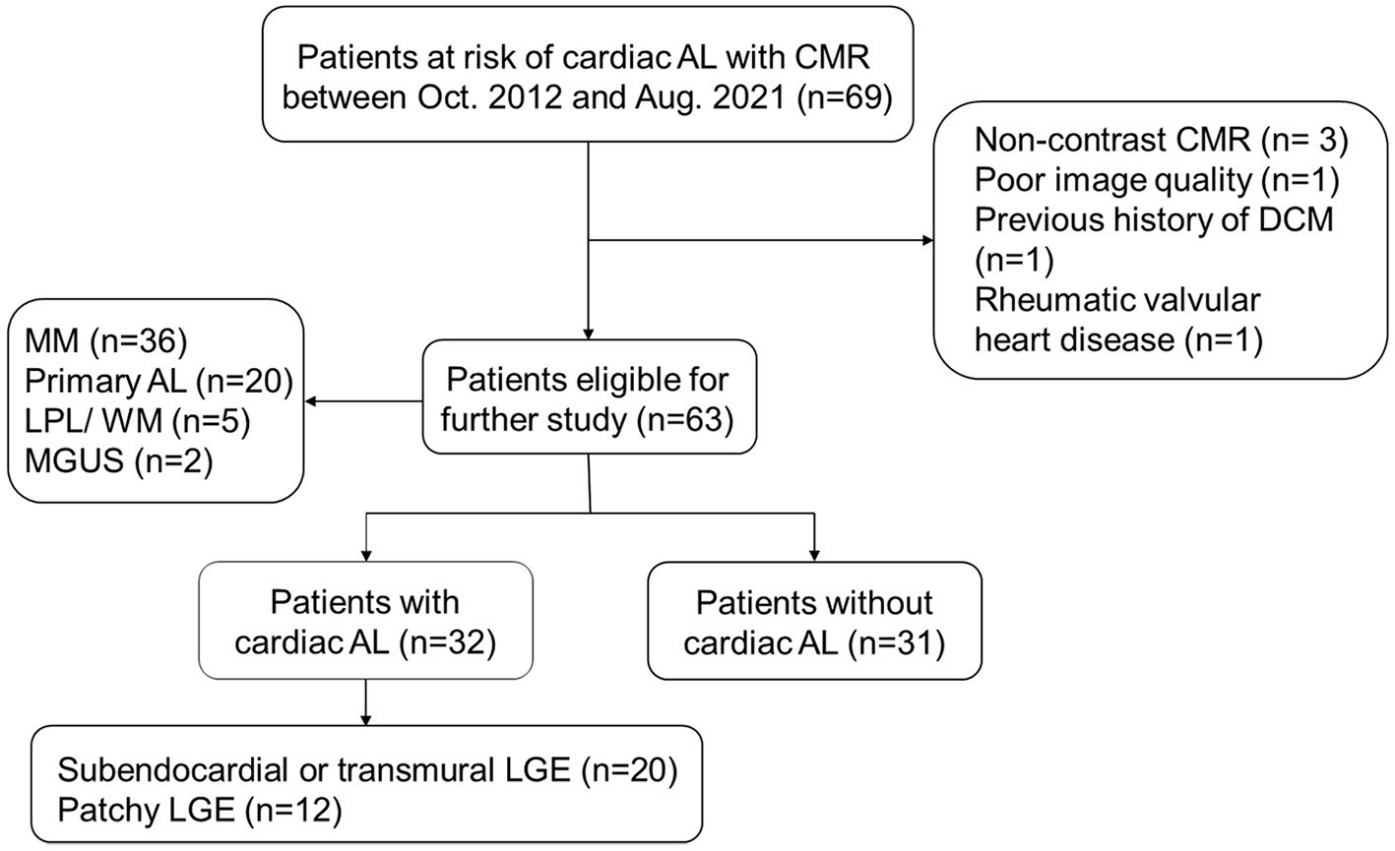
The flowchart of patient selection.

AL CA could occur in patients with diagnosed AL amyloidosis or other diseases with potential to proceeding into secondary AL amyloidosis, including multiple myeloma (MM), lymphocytic lymphoma (LPL)/Waldenstrom macroglobulinemia (WM), and monoclonal gammopathy of undetermined significance (MGUS) (14, 15). These patients would be defined as “at risk of AL CA” and undergo CMR scan when there were heart-related abnormalities in echocardiography, ECG, and lab tests (cardiac biomarkers) or when their clinicians considered there was necessity to exclude the presence of AL CA, especially in patients with proved systemic AL. Sixty-nine patients at risk of AL CA underwent CMR scan between October 2012 and August 2021 were initially integrated into our study (*n* = 69). Six patients met the following exclusion criteria were then excluded: Absence of LGE imaging (*n* = 3); poor image quality (*n* = 1); previous history of dilated cardiomyopathy (*n* = 1); coexistence with rheumatic valvular heart disease (*n* = 1). Ultimately, sixty-three patients were included (Figure 1). AL CA would be diagnosed if typical CA LGE pattern was observed. Typical LGE pattern was defined as: circumferential entire sub-endocardium involvement with different degree extension to surrounding myocardium, diffuse myocardial enhancement without myocardium nulling, or a scattering patchy pattern on LGE-imaging (14). Thirty-two patients were considered AL CA+ and thirty-one patients AL CA- according to the typical LGE criteria. Among AL CA+ patients, 18 (56%) patients had extra-cardiac biopsy proved amyloid deposition; 11 (34%) patients were with monoclonal protein identified by serum or urine immunofixation while extra-cardiac biopsy was negative or not performed in our hospital; 3 (9%) patients were with characteristic circumferential subendocardial LGE or diffuse LGE which could not be explained by other causes rather than CA.

Our study conformed to the declaration Helsinki and was approved by our institutional review board.

### Data Collection

Clinical data nearest to the CMR scan were collected through our electric history system, including baseline characteristics, lab results and 12-lead electrocardiogram (ECG) results. Low electrocardiographic QRS voltages (LQRSV) was defined as QRS amplitude of ≤10 mm in all the precordial leads or ≤5 mm in all standard limb leads (16). Left ventricular maximum wall thickness (LVMWT) and left atrial antero-posterior diameter (LAAPD) were measured on CMR image.

### CMR Scan Protocol and Image Analysis

All patients underwent CMR scan on 1.5 T clinical scanners (Signa Excite HD, GE, Boston, America; Avanto, Siemens Healthineers, Erlangen, Germany). Routine CMR scan protocol was described before (17). In brief, cine images of short axis, 2-chamber, 3-chamber, 4-chamber and outflow tract were acquired via steady-state free proceeding (SSFP) sequence (slice thickness = 8 mm, slice space = 2 mm, TR = 3.5 or 47.52 ms, TE = 1.5 or 1.11 ms, flip angle = 45° or 56°, Cardiac phases= 20 or 25). LGE image was obtained 8–10 min after the injection of 0.15 mmol·Kg^−1^ gadolinium (Beilu, Beijing, China). In addition, other routine sequences, including dark-blood T1, dark-blood T2 were also gained.

Routine function analysis as well as strain analysis was measured on CMR image, for part of functional data, like strain, were not routinely evaluated on echocardiography. CMR image post-processing was performed on a commercial post-processing software CVI 42 (Circle Cardiovascular Imaging; Calgary, Canada). The endocardium and epicardium of left ventricle were first contoured automatically by the software, and then adjusted by radiologists. Routine function parameters were acquired in Function SAX model, while strain parameters in Strain model. Global strain parameters were included for analysis, including global circumferential strain (GCS), global longitudinal strain (GLS) and global radial strain (GRS). We recorded GCS and GLS value with the absolute value for statistical analysis.

Presence of typical CA LGE pattern was independently determined by two radiologists with 3-year experience (Radiologist A) and 5-year experience (Radiologist B) in CMR diagnosis, respectively. When opinions of the two radiologists differed, a decision was reached by consensus or consultation with another more experienced investigator.

### Follow-Up

Patients were followed up for survival information through clinical electric history system or phone call. The primary end point was all-cause death. The final follow-up date was September 30, 2021. Follow-up duration was the time interval between the day of CMR scan and the day of death or study closure, whichever came first.

### Statistical Analysis

All statistical analyses were performed on SPSS 26.0 (IBM, Armonk, NY, USA). Continuous variables distributing normally were expressed as mean ± standard deviation (SD), otherwise they were expressed as median (inter-quartile). Categorical data were represented by frequencies and percentages. Student's *t*-tests or Mann-Whitney U test was performed for the comparison of continuous data between AL CA+ and AL CA- patients, while Fischer exact test was used to compare categorical data. All the continuous variables were transformed into categorical variables according to receiver operating characteristic (ROC) curve and cut-off value before logistic analysis (18). Univariable and multivariable logistic regression models were utilized to figure out independent predictors of AL CA. A risk score was constructed based on the predictors of AL CA and their corresponding regression coefficients. The score of every parameter was determined by the ratio of its β coefficient and the smallest β coefficient (19). The total score of individuals was calculated according to the established score. ROC curve was used to evaluating the accuracy of the diagnostic risk score in recognizing AL CA. Internal validation of this risk score was performed using bootstrapping method with 1,000 replicates on R version 4.0.5 (http://www.R-project.org). All patients were then grouped into high-risk group or low-risk group according to the diagnostic cut-off score. Kaplan-Meier plots and log-rank test were further used to test whether the risk score could provide extra prognosis information in addition to diagnostic value.

## Results

### Patient Characteristics

The baseline characteristics of patients were listed in Table 1. Our study finally consisted of 63 patients at risk of AL CA, including MM (*n* = 37), primary AL (*n* = 19), LPL/ WM (*n* = 5), and MGUS (*n* = 2). As shown in Table 1, no significant difference was found in age, sex, basic surface area (BSA), hypertension, and diabetes between AL CA+ and AL CA- group.

**Table 1:** Baseline characteristics of patients at risk of AL CA.

**Variable**	**All patients (*n* = 63)**	**AL CA+ Patients (*n* = 32)**	**AL CA- Patients (*n* = 31)**	***P***
Age, years	59 ± 10	59 ± 10	59 ± 10	0.998
Sex, male	40 (63%)	21	19	0.721
BSA, m^2^	1.68 ± 0.18	1.69 ± 0.22	1.68 ± 0.14	0.836
Hypertension	25 (40%)	11	14	0.382
Diabetes	2 (3%)	0	2	0.238
MM	37 (59%)	18	19	/
Primary AL	19 (30%)	12	7	/
LPL/WM	5 (8%)	1	4	/
MGUS	2 (3%)	0	2	/
NT-pro BNP	687 (104, 3,521)	2,524 (649, 4,614)	165 (58, 735)	<0.001
Elevated troponin I	12 (19%)	12 (38%)	0 (0%)	<0.001
Creatinine	76 (59, 94)	73 (58, 88)	80 (59, 124)	0.173
D-Dimer	0.46 (0.31, 1.18)	0.56 (0.33, 1.44)	0.45 (0.30, 0.92)	0.375
LQRSV	16 (25%)	12 (38%)	4 (13%)	0.041
Pericardial effusion	28 (44%)	23 (72%)	5 (16%)	<0.001
LVMWT, mm	13 (10, 17)	17 (14, 20)	11 (9, 13)	<0.001
LAAPD, mm	38 (34, 43)	39 ± 9	38 ± 6	0.796
LVEDV, ml	126 (100, 169)	124 (97, 163)	128 (109, 177)	0.216
LVESV, ml	52 (39, 92)	54 (39, 94)	50 (38, 79)	0.731
LVSV, ml	68 (53, 85)	62 (45, 78)	80 (62, 97)	0.005
LVEF, %	56 (46, 65)	52 (44, 59)	59 (50, 67)	0.020
GRS, %	19.8 (12.9, 28.9)	13.9 (8.6, 23.1)	28.1 (18.3, 34.1)	<0.001
GCS, %	15.1 ± 5.2	12.7 ± 5.1	17.6 ± 4.0	<0.001
GLS, %	8.5 ± 3.6	6.4 ± 2.5	10.6 ± 3.4	<0.001

*AL CA, cardiac light-chain amyloidosis; BSA, basic surface area; GCS, global circumferential strain; GLS, global longitudinal strain; GRS, global radial strain; LPL, lymphocytic lymphoma; LAAPD, left atrial antero-posterior diameter; LQRSV, low electrocardiographic QRS voltages; LVESV, left ventricular end-systolic volume; LVEDV, left ventricular end-diastolic volume; LVMWT, left ventricular maximum wall thickness; LVSV, left ventricular stroke volume; NT-pro BNP, N-terminal pro b-type natriuretic peptide; MGUS, monoclonal gammopathy of undetermined significance; MM, multiple myeloma; WM, Waldenstrom macroglobulinemia*.

### Lab Results and ECG Finding

N-terminal pro b-type natriuretic peptide (NT-pro BNP) in AL CA+ group is remarkably higher than that in AL CA- group [2,524 (649, 4,614) vs. 165 (58, 735) pg/mL, *P* < 0.001]. Since our institution used <0.01 rather than the exact value to record Troponin I when its level was lower than 0.01, we recorded Troponin I level as elevated or not elevated according to the recommended cut-off value of 0.11. In our study, elevated Troponin I was found in 12 AL CA+ patients while none AL CA- patients exhibited elevated Troponin I (*P* < 0.001). Creatinine and D-Dimer were not significantly different between AL CA+ and AL CA- patients (all *P* > 0.05).

Twelve AL CA+ and four AL CA- patients displayed low QRS voltage (LQRSV) on 12-lead ECG. The proportion of LQRSV between 2 groups were statistically different (*P* = 0.041).

### Morphology and Function

Pericardial effusion (PE) was more often observed in AL CA+ patients compared to AL CA- patients [23 (72%) vs. 5 (16%), *P* < 0.001]. LVMWT was thicker in AL CA+ patients than AL CA- patients [17 (14, 20) vs. 11 (9, 13) mm, *P* < 0.001]. LAAPD (39 ± 9 vs. 38 ± 6 mm, *P* = 0.796), left ventricular end-diastolic volume (LVEDV) [124 (97, 163) vs. 128 (109, 177) ml, *P* = 0.216] and left ventricular end-systolic volume (LVESV) [54 (39, 94) vs. 50 (38, 79) ml, *P* = 0.731] were similar between AL CA+ and AL CA- group. Left ventricular stroke volume (LVSV) in AL CA+ group was statistically lower than that in AL CA- group [62 (45, 78) vs. 80 (62, 97) ml, *P* = 0.005].

Left ventricular ejection fraction (LVEF) is the most common used parameter in the evaluation of cardiac function. In the comparison between AL CA+ and AL CA- patients, AL CA+ group had lower LVEF than AL CA– group [52 (44, 59) vs. 59 (50, 67)%, *P* = 0.020]. GRS [13.9 (8.6, 23.1) vs. 28.1 (18.3, 34.1)%, *P* < 0.001], GCS (12.7 ± 5.1 vs. 17.6 ± 4.0%, *P* < 0.001) and GLS (6.4 ± 2.5 vs. 10.6 ± 3.4%, *P* < 0.001) were significantly decreased in AL CA+ patients compared to AL CA- patients.

### Predictors of AL CA

The cut-off value and AUC of all continuous variables were listed in Table 2. Before logistic analysis, all continuous variables were transformed into dichotomous variable according to their corresponding best cut-off value. Table 3 showed the logistic analysis results. In the univariable analysis, the following parameters were with *P* value < 0.05: NT-pro BNP > 452 pg/mL, LQRSV, PE, LVMWT >13 mm, LVSV < 65.10 ml, LVEF < 55.89%, GRS < 15.14%, GCS < 15.88%, and GLS < 9.03%. In the multivariable analysis, independent predictors of AL CA were LQRSV (β = 3.26, OR = 26.06, *P* = 0.024), PE (β = 2.38, OR = 10.79, *P* = 0.049), LVMWT >13 mm (β = 4.51, OR = 90.56, *P* = 0.001), and GRS < 15.14% (β = 2.728, OR = 15.30, *P* = 0.038).

**Table 2:** AUC of all variables in differentiating AL CA+ patients from AL CA- patients.

**Variable**	**Cut-off value**	**AUC**	**Sensitivity**	**Specificity**	**Variable**	**Cut-off value**	**AUC**	**Sensitivity**	**Specificity**
Age, years	63	0.511	0.406	0.742	Pericardial effusion	yes	0.779	0.719	0.871
Sex, male	male	0.522	0.656	0.377	LVMWT, mm	13	0.893	0.781	0.903
BSA, m^2^	1.52	0.533	0.871	0.177	LAAPD, mm	31	0.501	0.903	0.219
Hypertension	yes	0.554	0.452	0.656	LVEDV, ml	98.43	0.591	0.903	0.281
Diabetes	yes	0.532	0.065	1.000	LVESV, ml	52.55	0.525	0.531	0.581
NT-pro BNP	452	0.777	0.844	0.710	LVSV, ml	65.10	0.708	0.313	0.258
Elevated Troponin I	yes	0.688	0.375	1.000	LVEF, %	55.89	0.670	0.645	0.656
Creatinine	83	0.600	0.484	0.719	GRS, %	15.14	0.810	0.935	0.594
D-Dimer	1.28	0.565	0.313	0.839	GCS, %	15.88	0.780	0.742	0.781
LQRSV	yes	0.623	0.375	0.871	GLS, %	9.03	0.835	0.742	0.844

*AL CA, cardiac light-chain amyloidosis; AUC, area under the curve; BSA, basic surface area; GCS, global circumferential strain; GLS, global longitudinal strain; GRS, global radial strain; LPL, lymphocytic lymphoma; LAAPD, left atrial antero-posterior diameter; LQRSV, low electrocardiographic QRS voltages; LVESV, left ventricular end-systolic volume; LVEDV, left ventricular end-diastolic volume; LVMWT, left ventricular maximum wall thickness; LVSV, left ventricular stroke volume; NT-pro BNP, N-terminal pro b-type natriuretic peptide*.

**Table 3:** Logistic analysis result for independent predictors of AL CA.

**Variable**	**Univariable analysis**				**Multivariable analysis**			
	**β**	**OR**	**95% CI**	***P***	**β**	**OR**	**95% CI**	***P***
NT-pro BNP	2.58	13.20	(3.86, 45.14)	<0.001				
LQRSV	1.40	4.05	(1.14, 14.43)	0.031	3.26	26.06	(1.54, 441.15)	0.024
Pericardial effusion	2.85	17.25	(4.69, 63.45)	<0.001	2.38	10.79	(1.01, 115.00)	0.049
LVMWT	3.51	33.33	(7.77, 142.97)	<0.001	4.51	90.56	(6.11, 1,341.73)	0.001
LVSV, ml	1.85	6.33	(2.11, 18.97)	0.01				
LVEF, %	1.24	3.47	(1.23, 9.78)	0.019				
GRS, %	3.05	21.19	(4.29, 104.67)	<0.001	2.728	15.30	(1.17, 200.03)	0.038
GCS, %	2.33	10.27	(3.21, 32.81)	<0.001				
GLS, %	2.74	15.53	(4.46, 54.09)	<0.001				

*AL CA, cardiac light-chain amyloidosis; CI, confidence interval; GCS, global circumferential strain; GLS, global longitudinal strain; GRS, global radial strain; LQRSV, low electrocardiographic QRS voltages; LVMWT, left ventricular maximum wall thickness; LVSV, left ventricular stroke volume; NT-pro BNP, N-terminal pro b-type natriuretic peptide; OR, odds ratio*.

### Construction of the Risk Score

**Figure 2:**
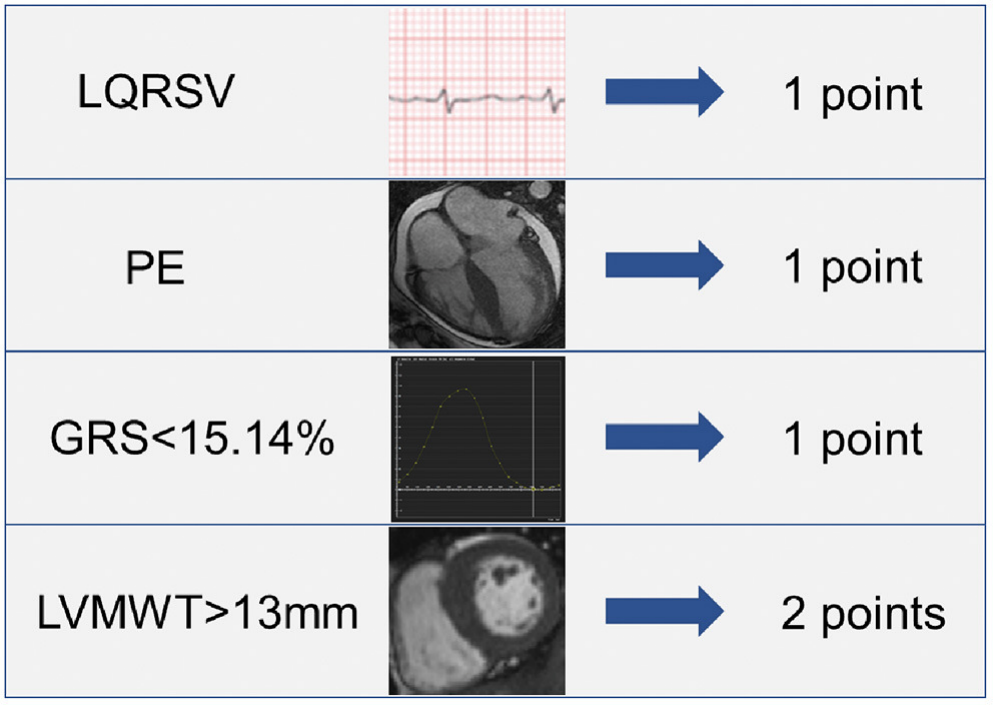
Details of the risk score.

The above-mentioned predictors of AL CA were assigned with point according to their β coefficients. As a result, the risk score incorporated LQRSV (1 point), PE (1 point), GRS < 15.14% (1 point), and LVMWT > 13 mm (2 points) was constructed (shown in Figure 2). The total score of individuals would be acquired by summing up the respective score of all the parameters.

### Diagnostic and Prognostic Value of the Risk Score

**Figure 3:**
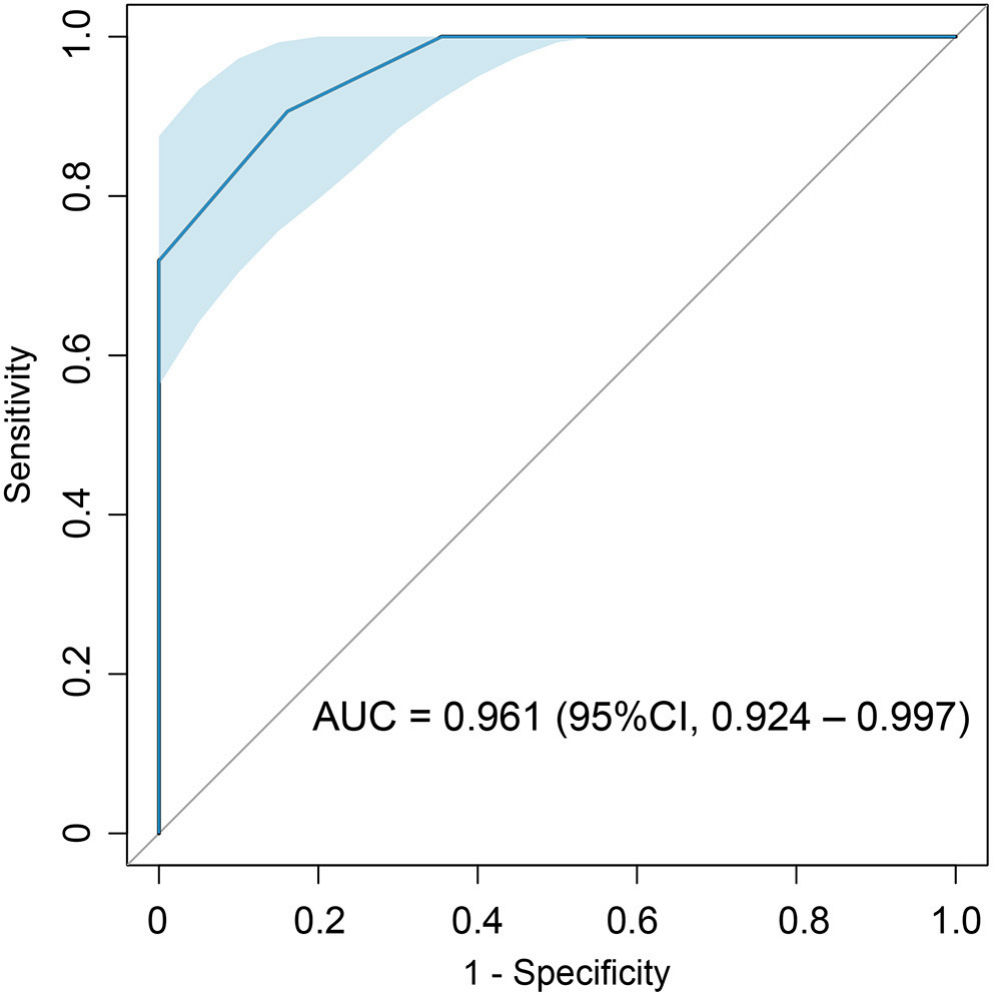
ROC analysis of the total score recorded by the risk score in identifying AL CA patients.

Performance of the risk score was evaluated by ROC curve. ROC curve showed that the total score with a cut-off point of 1.5 points were with highest AL CA diagnostic accuracy with an AUC of 0.961 (95% CI: 0.924–0.997, sensitivity: 90.6%, specificity: 83.9%) (Figure 3). Internal validation based on bootstrapping demonstrated an optimism-corrected AUC of 0.944 for the risk score.

**Figure 4:**
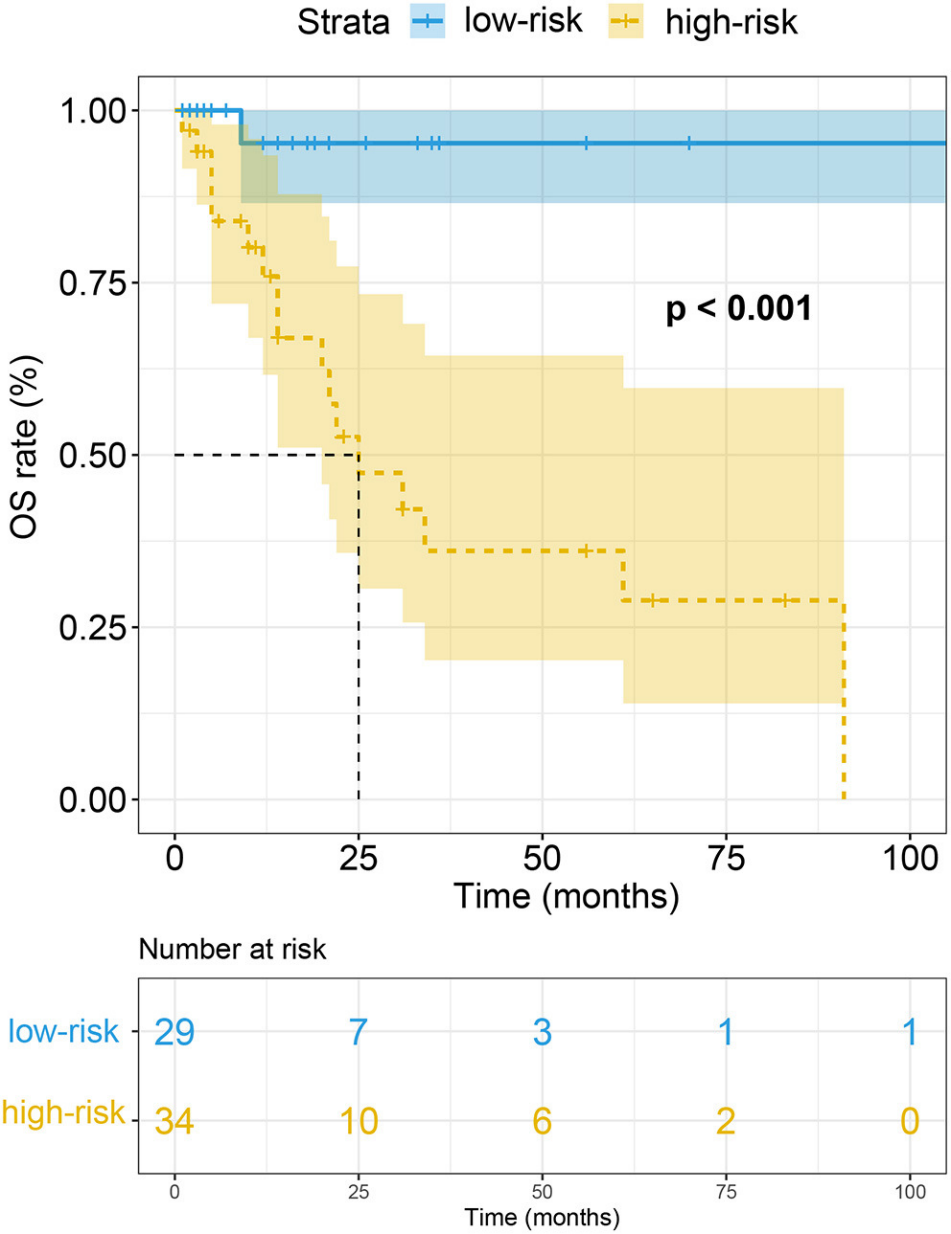
Kaplan-Meier curve of the overall survival rate in high-risk and low-risk group.

Patients at the risk of AL CA were divided into high-risk group (total score > 1.5 points) and low-risk (total score < 1.5 points) group using the diagnostic cut-off point. The median follow-up duration patients were 14 months [interquartile range (IQR): 6–24 months]. Eighteen patients including 14 AL CA+ patients and 4 AL CA- patients were dead during follow-up. Kaplan-Meier curve suggested that the high-risk group had poorer overall survival (OS) rate compared to the low-risk group. Log-rank test indicated the prognosis difference between high-risk and low-risk group was statistically different (*P* < 0.001) (Figure 4).

## Discussion

In our study, we construct a risk score helping diagnose cardiac involvement in patients at risk of AL CA. The risk score consists of four easily-acquired but important parameters: PE (1 point), LQRSV (1 point), CMR-derived impaired GRS (< 15.14%) (1 point) and increased LVMWT (>13 mm) (2 points). After summing up the score of individual, a total score larger than 1.5 points showed high performance in recognizing AL CA according to LGE criteria. On the basis of the established risk score, we further grouped all possible AL CA patients into high-risk group (total score > 1.5 points) and low-risk group (total point < 1.5 points). OS rate in high-risk group was remarkably lower than that in low-risk group.

Timely identification of cardiac involvement in patients at risk of AL CA is critical to the clinical management, whatever the primary cause of AL CA. For patients with primary systemic AL amyloidosis, cardiac involvement means higher early death rate after autologous stem cell transplant (ASCT), initial low dose of chemotherapy, poor tolerance for immunomodulatory drugs (IMiDs), and necessity to implement extra measures that concur to blood volume control (20). MM-associated AL amyloidosis, is the most important constituent of secondary AL amyloidosis. However, little is known about the appropriate management of MM-associated AL CA. Close surveillance and avoidance of treatment-related cardiac toxicity would contribute to delaying the progression of heart failure, leading to improved outcomes among MM patients with concurrent AL CA (21).

PE, LQRSV, CMR-derived impaired GRS and increased LVMWT are common abnormalities among AL CA patients (22). The presence of these abnormalities has an indication of suspected AL CA.

In AL CA patients, a PE of any size was more often observed with pleural effusion (23, 24). It is still not comprehensively known what the exact mechanism of PE formation is. Right ventricular failure during AL CA process can lead to the PE and this mechanism reasonably explains the common coexistence of pleural effusion in AL CA patients (23). Moreover, it has also been proposed that the presence of PE is the result of myocardial edema generally attributing to the amyloid infiltration and consecutive inflammation (25). When patients come to clinic for pericardial and pleural effusion with undetermined reason, it is necessary for clinicians to help their patient perfect relevant examinations in order to exclude the existence of AL CA.

Increased wall thickness following amyloid deposition is an important sign of cardiac involvement in patients with suspected AL CA. The degree of hypertrophy is associated with the degree of cardiac infiltration (26). The echocardiography criteria to diagnose AL CA is the left ventricular wall thickness >12 mm in the absence of any other causes of hypertrophy (27). In our study, the best cut-off value of LVWMT distinguishing AL CA+ patients from AL CA- patients is 13 mm. This value is a little higher than the established value. The disparity may be partially explained by the difference of inclusion patients. Hypertension was not listed as an exclusion criterion in our study. We acknowledged that hypertension can influence the wall thickness (28) even if hypertension was all well-controlled. Nevertheless, it is the real-world situation, considering the high prevalence of hypertension. Additionally, we measured LVWMT on CMR images, which is more reproducible than on echocardiography (29). The possible existed measure differences due to various imaging tools may lead to different results.

LQRSV is a common ECG finding in AL CA patients with reported incidence rate of 20 to 74% (30). Of note, LQRSV alone is not specific for AL CA. It can also be found among patients with other cardiac and extra-cardiac disease and even in the healthy (31). In our patients, there were 4 patients free of AL CA presenting LQRSV. Generally, patients with hypertrophic myocardium due to hypertension or hypertrophic cardiomyopathy (HCM) present high QRS voltage (HQRSV) abnormality on 12-lead ECG (32). Nevertheless, in AL CA patients, there is a contradiction phenomenon of the coexistence of hypertrophic myocardium and LQRSV (33). The difference of QRS voltage presentation between AL CA and other myocardial hypertrophic diseases can be explained by the different mechanism of hypertrophy. In patients with hypertension or HCM, presence of myocyte hypertrophy leads to HQRSV (34, 35). However, in AL CA patients, myocardial hypertrophy is caused by the extracellular amyloid deposition and no actual hypertrophic myocyte exists (36). Our study result is consistent with previous findings: If a patient with suspected AL CA has concomitant increased LVMWT (>13 mm) and LQRSV, the total score of this patient is 2 points, larger than the cut-off point 1.5. The risk score would indicate high likelihood of cardiac involvement in this patient.

Amyloid deposition in heart would impair cardiac function. LVEF has long been the cornerstone to measure cardiac function. However, in the early stage of cardiac involvement, only slight diastolic dysfunction exists, which cannot be detected by LVEF (37). Strain has been proved as a sensitive and robust marker of cardiac dysfunction in a series of clinical scenarios. It allows for a more direct function evaluation of left ventricle than traditional LVEF (38). GCS, GLS, and GRS are the major components of strain evaluation. GCS and GLS are respectively determined by the circumferential and longitudinal length change of myocardial fibers, while GRS describes the deformation of myocardial fibers to the center of the heart cavity, which reflects the thickness change of myocardial fibers (39, 40). GRS in our study is an independent predictor of AL CA. CMR-derived impaired GRS is the result of AL amyloid deposition. Abnormal amyloid aggregation in the myocardium would affect the thickening of myocardial fibers in cardiac cycle, through direct toxicity, ischemic impairment as well as increased ECV. A single center study with 60 healthy Chinese people indicated that the normal CMR-derived GRS value is 37.7 ± 9.6%. The best cut-off value of GRS differentiating AL CA+ and AL CA- patients according to our research was 15.14%, far below the normal value, indicating severe function impairment in AL CA patients.

In our study, the constructed risk score stressed the importance of overall assessment for clinical abnormalities in diagnosing AL CA. Indeed, single abnormality during clinical evaluation is not specific for AL CA, when considered separately. Our risk score would improve diagnostic confidence of clinicians by incorporating 4 common abnormalities rather than a single abnormality. Contrast CMR with LGE imaging is irreplaceable in the noninvasive evaluation of cardiac involvement among patients at risk of AL CA (10). Nonetheless, its clinical value is often restricted by its contraindications, like impaired renal function. There is high co-occurrence rate of renal amyloidosis in patients with CA (41). Therefore, though the risk score cannot take the place of the gold standard EMB, it can be clinically significant by promoting further confirming tests and even EMB, especially in patients with contraindications to contrast CMR.

In addition to the diagnosis of AL CA, prognosis information is also paramount to patients as well as their clinicians. The prognostic value of PE (24, 42), LQRSV (31), impaired strain (43, 44) and LVMWT (45) has been previously described alone. Nonetheless, to our knowledge, the joint prognostic value has not yet been discussed. The established risk score consists of these four parameters. Prognosis value investigation of this risk score partially allows for the combined prognostic evaluation of the four parameters. As expected, in patients at risk of AL CA, high total score is a poor prognosis marker. Since cardiac involvement has been widely proved to negatively affect the outcome among patients at risk of AL CA, it is plausible to speculate that patients with high risk of AL CA are more likely to suffer poor prognosis compared to those with low risk. Hence, the risk score has prognosis stratification value in patients with risk of AL CA.

There are a few inherent drawbacks of our study. First, our risk score was constructed on the basis of single center data with limited sample size. Though internal validation with bootstrapping method confirmed the performance of the risk score in recognizing AL CA, a multi-center prospective study is still necessary to validate our findings and construct a stable and widely applicated risk score. Second, patients who came for CMR scan may represent a status of relatively higher likelihood of cardiac involvement compared to those who did not. In addition, those without contrast CMR scan were excluded. Patient selection bias may exist. Third, both LVMWT and GRS were measured on CMR images rather than echocardiography. For patients unable to receive contrast CMR scan, echocardiography would be considered prior to Non-contrast CMR. However, Non-contrast CMR still keeps its superiority to echocardiography in the aspect of reproductivity and operator-independence. Further work is necessary to verify whether this risk score tool can be interchangeable between CMR and echocardiography. Fourth, in our study, we used typical LGE pattern rather than the gold standard EMB to diagnose AL CA. Though LGE is the visible result of amyloidosis deposition, it cannot be observed when the deposition is scarce (46). In addition, there were reported cases with coexistence of AL amyloidosis and cardiac transthyretin amyloidosis (ATTR CA) (47). Though the coexistence is rare, the lack of histological CA typing and gene sequencing would potentially make ATTR CA patients mistakenly included, further influencing the accuracy of this risk score tool.

## Conclusions

In conclusion, our study established a risk score based on PE, LQRSV, CMR-derived impaired GRS and increased LVMWT. When contrast CMR with LGE imaging is unavailable or contraindicated, this risk score is promising in early and accurately detecting cardiac amyloid deposition in patients at risk of AL CA. Furthermore, this risk score is also of prognostic value. For patients with high total score, different therapy and close follow-up should be considered. Future large-scale prospective study is warranted to validate our findings.

## Data Availability Statement

The raw data supporting the conclusions of this article will be made available by the authors, without undue reservation.

## Ethics Statement

The studies involving human participants were reviewed and approved by Ethics Committee of Sir Run Run Shaw Hospital. Written informed consent for participation was not required for this study in accordance with the national legislation and the institutional requirements.

## Author Contributions

YW and CP: conceptualization, data curation, and investigation. YW and WZ: formal analysis and methodology. HH and WZ: funding acquisition and resources. HH: project administration and supervision. WZ, CH, and JF: software. CH and JF: validation. JF: visualization. YW: writing-original draft. HH, CH, and JF: writing-review and editing. All authors contributed to the article and approved the submitted version.

## Funding

This work was funded by the National Natural Science Foundation of China (Grant No. 81873908, HH) and the Project Supported by Scientific Research Fund of Zhejiang University (XY2021032 and XY2021034).

## Conflict of Interest

The authors declare that the research was conducted in the absence of any commercial or financial relationships that could be construed as a potential conflict of interest.

## Publisher's Note

All claims expressed in this article are solely those of the authors and do not necessarily represent those of their affiliated organizations, or those of the publisher, the editors and the reviewers. Any product that may be evaluated in this article, or claim that may be made by its manufacturer, is not guaranteed or endorsed by the publisher.
